# Modulation of IL-6 induced *RANKL* expression in arthritic synovium by a transcription factor SOX5

**DOI:** 10.1038/srep32001

**Published:** 2016-08-23

**Authors:** Xiaoke Feng, Yumeng Shi, Lingxiao Xu, Qiuyue Peng, Fang Wang, Xiaoxi Wang, Wei Sun, Yan Lu, Betty P. Tsao, Miaojia Zhang, Wenfeng Tan

**Affiliations:** 1 Department of Rheumatology, the First Affiliated Hospital of Nanjing Medical University, China; 2 Department of Traditional Chinese Medicine, the First Affiliated Hospital of Nanjing Medical University, China; 3 Department of Cardiology, the First Affiliated Hospital of Nanjing Medical University, China; 4 Division of Rheumatology, UCLA School of Medicine, USA

## Abstract

Receptor activator of nuclear factor κB ligand (RANKL) is critically involved in bone erosion of rheumatoid arthritis (RA). We previously reported association between younger age at onset of RA and a *RANKL* promoter SNP that conferred an elevated promoter activity via binding to a transcription factor SOX5. Here we study the regulation of SOX5 levels in relation to *RANKL* expression in RA synovial fibroblasts (SF) and the development of bone erosion in the collagen-induced arthritis (CIA) mouse. Our data indicated SOX5 levels were higher in synovium and synovial fluid from RA compared to osteoarthritis patients. Pro-inflammatory cytokines upregulated *SOX5* and *RANKL* expression in both primary RA SF and the rheumatoid synovial fibroblast cell line, MH7A. Overexpression of SOX5 resulted in significantly increased *RANKL* levels, while knockdown of SOX5 resulted in diminished IL-6 mediated *RANKL* upregulation in MH7A cells. Chromatin immunoprecipitation (ChIP) showed approximately 3-fold enrichment of *RANKL*-specific DNA in anti-SOX5 immunoprecipitate in IL-6 treated MH7A cells as compared to untreated cells. Locally silencing *SOX5* gene significantly diminished RANKL positive cells and bone erosion in CIA mice. These findings suggest SOX5 is an important regulator of IL-6-induced *RANKL* expression in RA SF.

Receptor activator of nuclear factor κB ligand (RANKL) is critically involved in bone erosion of rheumatoid arthritis (RA) by enhancing osteoclast formation, function and survival^1^. Increased RANKL levels, locally and systemically, promote excessive osteoclast activity and bone resorption in RA^2^. Targeting RANKL by Denosumab, a fully human monoclonal anti-RANKL antibody, could efficiently prevent bone loss in RA^3^, highlighting the pivotal role of RANKL in mediating bone erosion.

RANKL is expressed on stromal/osteoblastic cells, chondrocytes, activated T, B cells, endothelial cells and synovial fibroblasts (SF)^4^. SF is the main source of RANKL in the inflamed joints of RA^4^. Inflammatory cytokines such as IL-6, IL-1β, TNF-α and IL-17, abundant in the synovial fluid and synovium, are potent inducers of *RANKL* expression in RA^4^. Binding of RANKL to its unique receptor RANK activates a broad range of signaling cascades, including NF-κB, MAPK and calcium signaling pathways that could mobilize a series of downstream osteoclast-specific transcription factors such as microphthalmia-associated transcription factor (MITF), c-Fos and nuclear factor of activated T cells c1 (NFATc1) to drive osteoclastogenesis and bone erosion^5^.

To date, how the inflammatory cytokines regulate *RANKL* gene transcription largely remains unknown. Moreover, although blockade RANKL by monoclonal anti-RANKL antibody Denosumab could efficiently prevent bone loss in resorptive diseases such as RA and osteoporosis, but also could cause rare but significant, clinical complications such as osteonecrosis of the jaw and atypical subtrochanteric femoral fractures^6^, which might due to profound inhibition of RANKL and subsequently affecting bone turnover. In this context, an understanding of how *RANKL* expression is regulated is therefore important to identify novel therapeutic targets for RA or other resorptive diseases.

We previously reported that a *RANKL* promoter single-nucleotide polymorphism (SNP) rs7984870 confers an elevated promoter activity after stimulation and is associated with younger age at onset of RA^7^,^8^. *In silico* analysis indicated the risk allele of this SNP might create a binding site to SOX5 in the *RANKL* promoter. We subsequently confirmed that SOX5 could bind to the promoter of *RANKL* gene in Jurkat T cell line after stimulation with TNF-α by electrophoretic mobility shift assay (EMSA)^7^, indicating a potential role of SOX5 in regulation inflammatory cytokine mediated *RANKL* expression.

SOX5 belongs to the SoxD group of Sox family transcription factor and could be expressed in multiple cell linages including the spermatids, neurons, fetal brain, striated muscles, chondrocytes, B cells^9^,^10^,^11^,^12^. SOX5 functions as transcription factors and primarily plays important roles in the regulation of embryonic development, cell fate, and chondrogenesis^13^. Elevated *SOX5* transcripts have been related to invasive growth and reduced apoptosis in tumors and have been associated with progression of certain cancers^14^,^15^,^16^. Recent studies have suggested the involvement of SOX5 in B-cell development^9^ and Th17 differentiation^17^, suggesting a role of SOX5 in immune responses. Here, we present our findings of the function of SOX5 in RA SF *in vitro* and in the experimental inflammatory arthritic mice *in vivo*, supporting this transcription factor SOX5 as an important regulator for cytokine-induced *RANKL* expression and the development of bone erosion in arthritic joints.

## Materials and Methods

### Patients and samples

Patients with RA or osteoarthritis (OA) and healthy controls (HC) were recruited from the First Affiliated Hospital of Nanjing Medical University. Synovial samples were collected from 20 RA and 20 OA patients undergoing knee arthroplasty. The diagnosis of RA conformed to American College of Rheumatology 1987 revised criteria^18^. Synovial fluid samples were collected from 30 active RA (defined as DAS28 scores >3.2 before the initiation of disease-modifying anti-rheumatic drugs [DMARDS] treatment) and 27 OA patients (recruited according to American College of Rheumatology knee OA criteria)^19^. Clinical characteristics of RA and OA patients are listed in Table S1. The use of human materials was approved by the Institutional Review Board of the First Affiliated Hospital of Nanjing Medical University (Permit Number: Jsph-EC201303114), and written informed consent was obtained from all individuals before the operative procedure. The animal experiments were conducted with the approval of Institutional Animal Care and Use Committee of Nanjing Medical University (Permit Number: IACUC-2014060206). All experiments were carried out in accordance with the approved guidelines.

### Cell culture

Synovial tissues obtained from participating patients were cut into small pieces and digested with collagenase, and SF was identified by flow cytometry as previously described^20^,^21^. SF between 3 to 8 passages were used for experiments. SF were treated with IL-1β (100 ng/ml, Peprotech, 1-11B), IL-6 (100 ng/ml, Peprotech, 500-P56), TNF-α (100 ng/ml, Peprotech, 315-01A) or IL-17 (100 ng/ml, Peprotech, 200-17) for 24h to investigate effects of inflammatory cytokines on *SOX5* and *RANKL* expression. For gene transfection experiments, the human rheumatoid fibroblast-like synoviocyte MH7A cell line was a generous gift from Dr. Seiichi Tanuma (Tokyo University of Science)^22^.

### Immunohistochemistry and Immunofluorescence microscopy

Synovium from patients or mice was snap-frozen and embedded in paraffin for immunohistochemstry or immunofluorescence staining as we previously reported^23^. Rabbit anti–SOX5 (Santa Cruz, sc-20091), mouse anti-RANKL (Abcam, ab22113) and rabbit anti–TRAP (Abcam, ab96372) were used as the primary antibodies for immunohistochemistry. For double-immunofluorescence labeling of human joint tissues, the following primary antibodies were used: rabbit anti–SOX5, mouse anti-TRAP, mouse anti-FGF2 (Abcam, ab8880), mouse anti-CD68 (Abcam, ab125212) and mouse anti-RANKL. Using fluorescence microscopy, cell types were distinguished according to their characteristic morphology and confirmed by immunoreactivity with anti-FGF2 (FLS), anti-CD68 (macrophages) and anti-TRAP (osteoclast) antibodies.

### Confocal Microscopy

MH7A were treated with or without IL-6 (100 ng/ml) for the indicated time interval and fixed with 100% methanol and then washed with PBS and incubated with the rabbit polyclonal SOX5 antibody and/or the mouse monoclonal RANKL antibody. Cells were washed with PBS and incubated with rabbit anti-mouse IgG-Rhodamine conjugated or goat anti-rabbit IgG-FITC conjugated. Confocal microscopy was performed on an LSM-510 laser scanning microscope (Olympus, Center Valley, PA). Final images were processed using Adobe Photoshop software.

### Construction of shSOX5, lentivirus-shSOX5 and recombinant Adenovirus *SOX5* expression vectors

For construction of shSOX5, two different siRNA sequences that silenced *SOX5* effectively were used in this study (Table S2). The complementary DNA oligonucleotides were subcloned into the hairpin siRNA expression vector pRNAT-U6.1/Neo (Invitrogen). For local silencing *SOX5* in CIA mice joint, the recombinant lentiviral vector hU6-MCS-CMV-eGFP was used to construct lentivirus-SOX5 shRNA as previously described^24^. For construction of *SOX5* overexpression vector, the full-length of *SOX5* cDNA was generated via standard PCR and inserted into the pAd- CMV-MC5 as described^24^. All the cloned sequences were confirmed by DNA sequencing.

### Overexpression and knockdown experiments

For silencing *SOX5*, 1 μg/ml of shSOX5 was transfected into MH7A for 24 h using X-treme GENE HP DNA Transfection Reagent (R&D, 6365787001) and then treated with IL-6 for 24 h. Non-targeting shRNAs served as negative controls (described as mock in the current study). For SOX5 overexpression experiments, MH7A was transduced with 10^7^pfu/ml of Ad-SOX5 for 96 h. Control transfectants were obtained by transduction with Ad-EGFP.

### Chromatin immunoprecipitation (ChIP)

ChIP assays were performed essentially as described^25^. In brief, MH7A were treated with or without IL-6 (100 ng/ml) for 24h and cross-linked with 1% formaldehyde for 10 minutes and then lyzed inlysis buffer containing with protease inhibitor (Millipore). DNA was fragmented into ~200bp pieces using a Branson 250 sonicator. Each ChIP sample containing 100 μg nucleoprotein was used for the immunoprecipitation reaction with anti-SOX5 and nonspecific IgG, and 10% of the pre-cleared chromatin was set aside as input control. Purified DNAs were subjected to quantitative PCR (qPCR) using the primer sets listed in Supplementary Table S3. Fold enrichment of the targeted genomic sequences were calculated over IgG.

### Real-Time PCR and Western Blotting

Gene expression was quantified by SYBR Green real-time PCR using an ABI Prism 7900 Sequence Detection System. The sequences of the primers are listed in Table S3. Relative expression was calculated with normalization to β-actin values by using the 2−ΔΔct method. The following antibodies were used for Western blotting: rabbit polyclonal anti-SOX5 antibody (Millpore, ABN513); goat anti-rabbit RANKL antibody (Abcam); rabbit polyclonal anti-β-Actin antibody (Cell Signaling Technology, 8H10D10).

### Induction of collagen-induced arthritis in DBA/1J mice and histological assessment

The induction of CIA was described previously^26^. Briefly, male 6 wk-old DBA/1J (purchased from purchased from the Shanghai Laboratory Animal Center, Chinese Academy of Science) mice were injected intradermally at the base of the tail with 200 μg of bovine CII (Chondrex, 20021) emulsified with complete Freund adjuvant, and their arthritis manifestations were graded and scored as described^26^. For standard histologic assessment, hind paws were fixed overnight in 4% paraformaldehyde and decalcified using EDTA. The material was then embedded in paraffin, cut and 1–2 μm sections were stained with H&E. The histological arthritis score was determined in a blinded fashion for changes in synovial lining, cellular infiltrate, cartilage damage, and pannus, as previously described^27^.

### ELISA

Concentrations of SOX5 levels in synovial fluids of RA and OA patients were quantified using a commercial ELISA kit (Huamei, Wuhan, China, 20110647) according to the manufacturer’s recommendations. Briefly, synovial fluids samples (1: 5 dilution) and standards were added to the 96-well plates. After incubation for 2 hours and washing 5 times, mouse SOX5 conjugate was added, followed by incubation with TMB substrate solution and stop solution. The intensity of the color reaction was measured by a microplate reader at a wavelength of 450 nm. Concentrations of SOX5 were determined by a standard curve according to the manufacturer’s instructions.

### Micro-computed tomography (CT) analyses

Computed tomographic images of the knee joints and paws of the mice in all three groups (n = 5) were acquired on day 42, using a micro-CT Scan SkyScan1176S scanner at a resolution of 9 μm. Image acquisition required 6 min using the following parameters: 50 kV, 500 μA, 0.5 mm aluminium filter, 180° scan, rotation step 0.7° and frame averaging of 1. For verification of bone destruction, 3-dimensional models of the knee joints and paws were reconstructed using SkyScan CT Analyzer version 1.8.

### Osteoclast differentiation and TRAP staining

To assess the effect of SOX5 on osteoclastogenesis, RAW264.7 cells were plated at a density of 2 × 10^4^ cells/well in a 12-well culture plate, and transfect with mock or Lv-siSox5 for 48 h and then cultured with or without 100 ng/ml RANKL plus 20 ng/ml M- M-CSF; After 3–5 d, cells were stained for TRAP using an Acid Phosphatase, Leukocyte (TRAP) Kit (Sigma) according to the manufacturer’s instructions. TRAP-positive multinucleated cells with three or more nuclei were counted as osteoclasts under a light microscope.

### Statistical analysis

Data were presented as mean and SD. Data were firstly tested for conformation to a normal distribution using the Shapiro–Wilk test and then were analyzed by Student’s t test (pair-wise comparisons) or analysis of variance (ANOVA), as appropriate. P values less than 0.05 were considered significant.

## Results

### Increased expression levels of SOX5 in RA synovium

**Figure 1:**
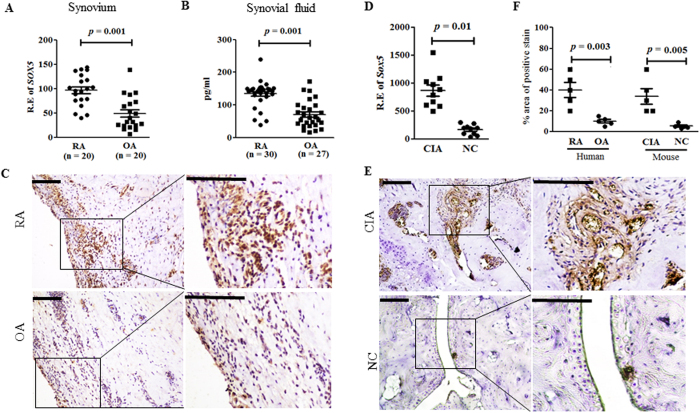
Increased expression levels of SOX5 in RA synovium. (**A**) Real-time-PCR analysis of *SOX5* expression in synovium from RA (n = 20) and OA (n = 20) patients. (**B**) ELISA analysis of SOX5 levels in synovial fluid from RA (n = 30) and OA (n = 27) patients. (**C**) Representative images of RA (upper) and OA (lower) synovium immunostained for SOX5 (n = 5, respectively). (**D**) Real-time PCR analysis of *Sox5* expression in arthritic joint of CIA mice and normal control (NC) mice (n = 10 mice per group). (**E**) Immunohistochemical detection of SOX5 expression in the synovium from CIA (upper) and NC (lower) mice (n = 5, respectively). (**F**) Percentage of SOX5 positive area in synovium from human RA, OA, CIA mouse and NC. Values are means ± SD. Scale bar, 100 μm. R.E, Relative expression.

We previously reported that SOX5 could bind to the promoter of *RANKL* gene in Jurkat T-cells after stimulation^7^. To explore possible functions of SOX5 in RA pathogenesis, we firstly compared the levels of SOX5 expression between RA and osteoarthritis (OA) patients. Real-time PCR revealed higher expression levels of *SOX5* relative to *β - actin* in synovium of RA patients (n = 20) than those in OA counterparts (n = 20) (Fig. 1A, *p* = 0.001). We further examined protein levels of SOX5 in synovial fluids from RA (n = 30) and OA patients (n = 27). Similar to the expression in synovium tissue, SOX5 was increased approximately 2-fold in RA synovial fluids (Fig. 1B, *p* = 0.001) compared with those in OA patients. Immunohistochemical staining indicated SOX5 positive cells were more abundant in RA synovium samples than those of OA samples (n = 5 each) (Fig. 1C,F).

We extended these results using the CIA DBA/1J mice. Consistent with the expression in human RA synovium, real-time PCR (Fig. 1D) and immunohistochemical staining (Fig. 1E,F) revealed higher expression of Sox5 in inflamed synovium from CIA mice as compared with normal control mice (NC).

### Pro-inflammatory cytokines induce SOX5 and RANKL expression in RASF

**Figure 2:**
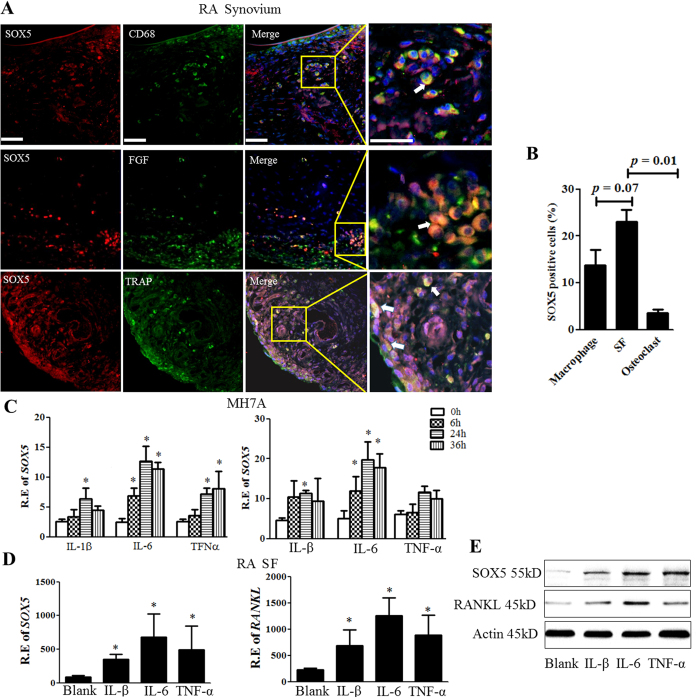
SOX5 is up-regulated by pro-inflammatory cytokines in SF of RA synovium. (**A**) Representative immunofluorescence microscopy images of SOX5 and macrophage marker CD68 (upper), SF marker FGF (middle) or osteoclast marker TRAP (lower) in RA synovium. Scale bar, 100 μm. The arrows point at double-stained cells. (**B**) The percentage of macrophage, SF and osteoclast positive for SOX5staining. (**C**) MH7A cells were treated with IL-1β (100 ng/ml), IL6 (100 ng/ml), or TNFa (100 ng/ml) for 0–36 h. SOX5 (left) and RANKL (right) expression were quantified by Real-time PCR (n = 3). (**D**) Primary cultured SF were treated IL-1β (100 ng/ml), IL6 (100 ng/ml), or TNFa (100 ng/ml) for 24 h, and SOX5 (left) and RANKL (right) mRNA levels were quantified (n = 6). Values are means ± SD (*p < 0.05). (**E**) Representative images of western-blot detection of SOX5 and RANKL protein expression of in MH7A after IL-1β, IL6, or TNFa treatment for 24 h. All experiments were performed in triplicate and were repeated three times.

Given that SOX5 was up-regulated both in lining cells and sublining cells of synovium in RA patients (Fig. 1C), which primarily consists of SF and macrophages, we determined which cell type expresses SOX5 in inflamed RA synovium. Double-immunofluorescence studies were used to investigate co-localization of SOX5 and markers for macrophages (CD68), SF (FGF) and osteoclasts (TRAP). More SOX5-positive cells could be detected in SF (Fig. 2A, middle and Fig. 2B) than in macrophages (Fig. 2A, upper and Fig. 2B), whereas co-localization between TRAP positive cells and SOX5 was only occasionally observed in RA synovium (Fig. 2A, lower and Fig. 2B). These data indicate that SOX5 is mainly expressed in SF of RA synovium.

Next, we investigated the mechanisms that could induce high expression of SOX5 in RA SF. Human rheumatoid fibroblast-like synoviocyte MH7A cell line were treated with inflammatory cytokines IL-1β, TNF-α, IL-6 or IL-17 for 0–36 h. Real-time PCR indicated that IL-1β, TNF-α and IL-6 could induce up-regulation of *SOX5* in MH7A cell line at 24–36h (Fig. 2C, left). As expected, the expression levels of *RANKL* were concomitantly increased in MH7A (Fig. 2C, right) in response to the pro-inflammatory cytokines. Among these cytokines, IL-6 showed the robust effect on inducing *SOX5* and *RANKL* expression at 24h, whereas IL-17 failed to drive *SOX5* expression (data not shown). The similar results were confirmed at primary RA SF upon these inflammatory cytokines stimulation for 24 h (Fig. 2D, left for *SOX5* and right for *RANKL* expression. Consistent with mRNA expression, western blot analysis showed that the protein levels of SOX5 and RANKL were concomitantly increased in MH7A after stimulation (Fig. 2E). These findings suggest that inflammatory cytokines upregulate SOX5 in SF, which is accompanied by increased RANKL levels.

### Co-localization of SOX5 and RANKL in RA SF

**Figure 3:**
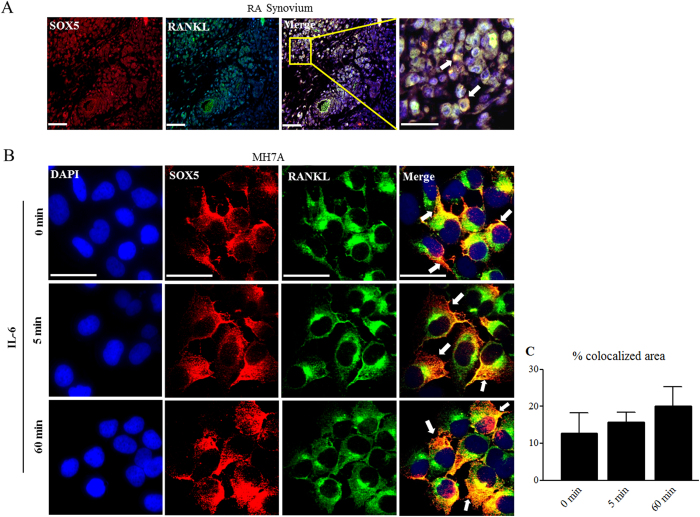
Pro-inflammatory cytokines promote the interaction of SOX5 with RANKL. (**A**) immunofluorescence microscopy used to identify co-staining of SOX5 and RANKL in RA synovium. Arrows indicate SOX5 and RANKL double-positive. Scale bar, 100 μm. (**B**) Confocal microscopy indicated that IL-6 enhances apparent SOX5 co-localization with RANKL in MH7A. Representative laser-scanning confocal micrographs demonstrate the distribution of SOX5 (red), RANKL (green) and overlay (yellow). (**C**) Quantitative analysis of colocalized area of SOX5 and RANKL by comparing the colocalized area to the total immunofluorescent area. Scale bar, 100 μm. All experiments were performed in triplicate and were repeated three times.

We further examined SOX5 and RANKL expression in synovial tissue from RA patients by immunofluorescence staining. We found that SOX5 co-localized with RANKL in the inflamed RA synovium (Fig. 3A). We further assessed the effect of pro-inflammatory SOX5 and RANKL expression in RA SF by immunofluorescence staining. Our results (Fig. 2C,D) indicated that IL-6 was the strongest inducer of SOX5 and RANKL expression in SF at 24 h, and then we confirmed this result at 48–72 h (data not show). So, IL-6 was used to stimulate MH7A cells in the next experiment. Although did not reach a statistical differences, we found an increased tendency of co-localization of SOX5 and RANKL on MH7A cell surface at 0 to 60 min after IL-6 stimulation by confocal microscopy (Fig. 3B,C), implying IL-6 could prompt co-localization of SOX5 and RANKL in RA SF.

### SOX5 knockdown by shRNA reduce IL-6 -mediated *RANKL* expression in RA SF

**Figure 4:**
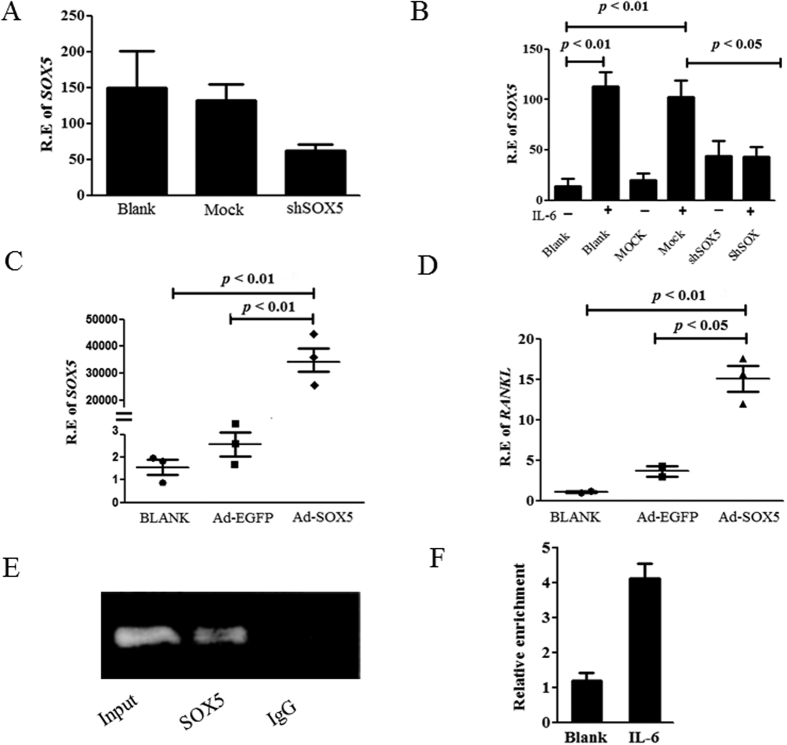
*RANKL* mRNA expression in *SOX5* knockdown or overexpression MH7A. (**A**) Expression of *SOX5* in SOX5-shRNA transfected MH7A. Values are means ± SD. (**B**) IL-6 induced *RANKL* expression in SOX5-shRNA treated or mock-transfected MH7A. Values are means ± SD. (**C**) Expression of *SOX5* in MH7A after Ad-SOX5 transfection. (**D**) *RANKL* expression in Ad-SOX5 or Ad-EGFP transfected (control) MH7A. Values are means ± SM. Blank in Figure A to D represents non-virus infection. (**E**) A representative gel of the amplified RANKL DNA immunoprecipitated with SOX5 antibodies in ChIP assay. Rabbit IgG served as negative control. (**F**) qPCR analyses SOX5 immunoprecipitates for the enrichment of RANKL-specific DNA in IL-6 treated MH7A cells. Fold enrichment of RANKL containing sequences in SOX5 ChIP was determined by comparing enrichment with IgG. All experiments were performed in triplicate and were repeated three times.

We assessed the effect of *SOX5* knockdown on IL-6 induced *RANKL* mRNA expression in RA SF using shRNA targeting *SOX5*. MH7A was transfected with SOX5-shRNA to silence *SOX5* expression. As shown in Fig. 4A, SOX5-shRNA transfection resulted in an approximately 51% decrease in *SOX5* expression by MH7A cells, compared with mock transfected group. Consistent with Fig. 2D, *RANKL* expression was markedly increased in MH7A upon IL-6 stimulation. However, IL-6-induced *RANKL* expression was significantly downregulated in SOX5-shRNA treated MH7A in comparison with mock-transfected controls (Fig. 4B, *p* < 0.05), suggesting that SOX5 is required for IL-6-induced *RANKL* expression in RA SF.

### Overexpression of SOX5 regulates expression of RANKL in RA SF

We asked whether SOX5 could directly regulate *RANKL* expression by overexpressing *SOX5* in MH7A. As shown in Fig. 4C,D, transfection with adenovirus vectors expressing *SOX5* (Ad-SOX5) resulted in overexpression of *SOX5* in MH7A cells with concomitantly increased *RANKL* levels (Fig. 4D, p < 0.001), comparing to those transfected with EGFP-expression vector controls. Osteoprotegerin (OPG) is a decoy receptor for RANKL. The levels of OPG was also determined in SOX5 overexpressed and silenced MH7A cells. However, transfection with Ad-SOX5 or SOX5-shRNA did not affect OPG expression in MH7A (Figure S1). Taken together, these findings strongly suggest that SOX5 plays an important role in the regulation of *RANKL* expression.

### IL-6 facilitates the binding of SOX5 to RANKL promoter in SF

We have previously shown that SOX5 could bind to genomic sequences within the *RANKL* promoter in Jurkat cells using EMSA assay; moreover, luciferase study indicated *RANKL* promoter activity could be markedly decreased after mutation of SOX5 binding site (−1857) in *RANKL* promoter, implying SOX5 might function as an activator of the human *RANKL* promoter^7^. To further elucidate interaction between the transcriptional factor SOX5 and *RANKL* promoter, a ChIP assay was performed. A markedly higher amount of chromatin containing *RANKL* promoter region was immunoprecipitated by anti-SOX5 antibody compared with control IgG in RA SF (Fig. 4E). We then addressed whether IL-6 stimulation could promote the binding of SOX5 with *RANKL* in SF. We analyzed SOX5 immunoprecipitates by qPCR for the enrichment of *RANKL*-specific DNA in IL-6 treated MH7A cells. As shown in Fig. 4F, MH7A treated with IL-6 induced approximately 3-fold enrichment of *RANKL*-specific DNA in anti-SOX5 immunoprecipitate as compared to untreated cells. These results indicate that IL-6 could facilitate the binding of SOX5 to *RANKL* promoter in SF.

### Intra-articular administration of Lentivirus expressing shRNA for *Sox5* ameliorates bone erosion in CIA mice in part by suppression *Rankl* expression

**Figure 5:**
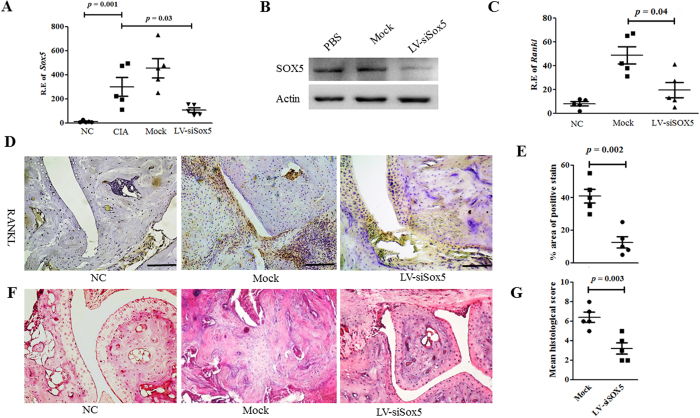
Lentivirus-mediated *Sox5* gene silencing ameliorated clinical symptoms and bone erosion in CIA mice. (**A**) Real–time PCR analysis of *Sox5* expression in arthritic joint from NC, CIA, mock siRNA and LV-siSox5 treated CIA mice at day 24 after the second immunization (n = 5, per group). (**B**) Western blot analysis of Sox5 levels in the joint tissue of immunized mice 24 days after treatment with LV-siRNA or Mock siRNA. (**C**) Real-time-PCR analysis of *Rankl* expression in the synovium from LV-siSox5 or Mock siRNA treated CIA mice. (**D**) Immunohistochemical detection of RANKL positive cell expression in synovium samples from CIA mice after treatment with LV-siSox5 or Mock siRNA. (**E**) Percentage of SOX5 positive area in synovium from mock and LV-siSox5 treated CIA mice. (**F**) H&E histological analysis of representative ankle sections of normal and CIA mice with intra-articular injection of LV-siSox5 or Mock siRNA at day 24 after the second immunization. (**G**) Histological scores of ankle sections shown at day 24 after the second immunization (n = 5). Scale bar, 100 μm. Values are means ± SM.

We demonstrated the presence of increased SOX5 in RA synovium and that SOX5 is critical for IL-6-induced *RANKL* expression *in vitro*. Next, we investigated the possible *in vivo* functions of Sox5 in the CIA model. We locally knocked down *Sox5* expression in CIA mice by injecting lentiviral vectors expressing siRNA (Lv-siSox5) intra-articularly into the hind ankle at day 1 of the second immunization. Administration of Lv-siSox5 into joints of CIA mice effectively decreased *Sox5* mRNA in the joint tissue as compared with mock siRNA injection (Fig. 5A, *p* = 0.03). Consistent with mRNA expression, Sox5 protein level was significantly decreased in the joint tissue from Lv-siSox5-treated CIA mice (Fig. 5B) in comparison with PBS treated or mock siRNA transfection.

CIA incidence was decreased by almost 50% after Lv-siSox5 treatment (Figure S2A); accordingly, the arthritis score (Figure S2B) and articular swelling (Figure S2C) were significantly decreased. Consistent with the inhibitory effects of *RANKL* expression *in vitro*, local silencing of *Sox5* markedly decreased *Rankl* mRNA expression (Fig. 5C, *p* = 0.04) in the arthritic joint from CIA mice. Immunohistochemstry staining indicated that *RANKL* protein expression were diminished in synovium samples from Lv-siSox5-treated CIA mice (Fig. 5D,E) compared with mock–treated mice. Histological analysis (Fig. 5F) indicated that local silencing of *Sox5* in joint tissues significantly inhibited synovitis, synovial hyperplasia and bone erosion in CIA mice.

**Figure 6:**
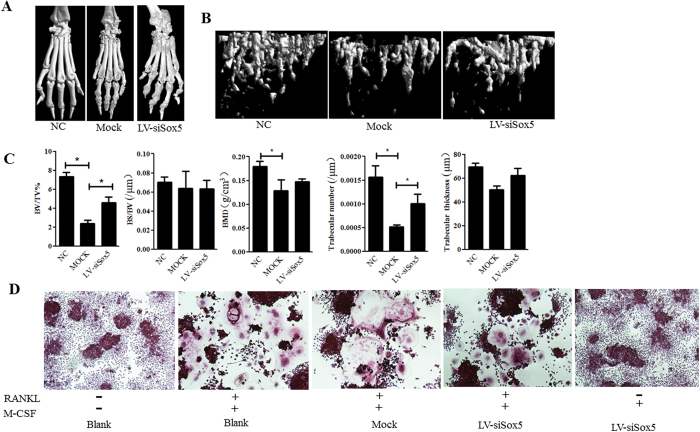
Bone erosion in the joint of *Sox5* gene silencing CIA mice. (**A**) Representative CT scan photographs of hind paws at day 24 after the second immunization. Scale bar, 100 μm. (**B**) Representative longitudinal 3D micro-CT images showing changes in trabecular bone microarchitecture regions from LV-siSox5 or Mock siRNA treated CIA mice. (**C**) Structural parameters in LV-siSox5 or Mock siRNA treated CIA mice. (**D**) Osteoclast formation in osteoclast precursor cell line RAW264.7 transfected with Lv-siSox5 or mock in presence and absence of RANKL. Values are means ± SD. BV/TV: Percent bone volume; BS/BV: Bone surface/volume ratio; BMD: bone mineral density.

Micro-CT confirmed a significant decrease in bone loss in the periarticular bone of paws and ankles from Lv-siSox5 treated CIA mice (Fig. 6A,B). We quantified the bone microarchitecture values of bone volume, trabecular thickness, trabeculae number and bone mineral density. Levels of bone volume, trabeculae number and bone mineral density was statistically significant decreased in Mcok treated CIA mice. However, bone volume, and trabeculae number and bone mineral density was increased in Lv-siSox5 treated CIA mice as compared with Mock treated mice (Fig. 6C).

Osteoclast is the unique cell that responsible for bone erosion in RA. We addressed whether SOX5 can directly stimulate osteoclast formation independent of RANKL. Osteoclast precursor cell line RAW264.7 was transfected with Lv-siSox5 for 48h and then were treated with 150 ng/ml RANKL and 30 ng/ml M-CSF for 3–5 d to induce osteoclast differentiation. Silencing Sox5 did not affect the formation of TRAP-positive cells in osteoclast precursor cell treated with RANKL. However, the multinucleated TRAP staining positive osteoclast-like cells were not found in Lv-siSox5 transfected RAW264.7 in the absence of RANKL, implying that SOX5 mediated bone erosion is depended of RANKL (Fig. 6D). Collectively, these results indicate that local suppression *Sox5* expression ameliorates arthritis and bone loss in CIA mice likely by inhibiting *Rankl* expression.

## Discussion

It is well established that the bone damage in RA is caused by inflammation-induced abnormal expression of RANKL to drive osteoclastogenesis^2^. The downstream signals required for RANKL activation and osteoclast differentiation have been extensively studied^28^. However, there is limited understanding of how the pro-inflammatory cytokines regulate transcription of the human *RANKL* gene^28^. Our current findings identify SOX5 as an important regulator of proinflammatory cytokine-induced expression of RANKL in RA. To our knowledge, this is the first study providing evidence for high expression of SOX5 in synovium samples of RA patients and CIA model. Our data demonstrate that knockdown of *SOX5* abrogated the stimulatory effects of IL-6 on *RANKL* expression in SF *in vitro*. Local *Sox5* gene silencing significantly ameliorated clinical symptoms and bone erosion in CIA mice, which is likely mediated by suppressing *Rankl* signaling. Collectively, these results provide novel insights into the regulation of RANKL expression in inflamed synoviocytes of RA patients by SOX5.

SOX proteins predominantly function as transcriptional activators by differential binding to particular flanking sequences of target genes or by their interactions with other transcription factors^28^. To date, little is known about SOX5 in RA. Our previous EMSA experiment showed that SOX5 could bind to *RANKL* promoter in Jurkat cells after stimulation with TNFα^7^, providing direct evidence of an interaction between SOX5 and RANKL. In current study, we validated that SOX5 was able to directly bind to the promoter of *RANKL* by ChIP assay. We determined the direct effect of SOX5 on *RANKL* transcript in RA SF. We found *in vitro* knockdown the *SOX5* significantly decreased IL-6-induced *RANKL* expression in SF, implying that SOX5 is required for pro-inflammatory induced *RANKL* expression.

Importantly, our data indicated that SOX5 only showed weak binding activity with *RANKL* in unstimulated SF; however, this binding activity was markedly enhanced after IL-6 treatment in the RA synovium derived cell line, MH7A in our ChIP assay. This result is reinforced by the concomitantly increased SOX5 and RANKL levels in primary RA SF and MH7A cell line after stimulation with IL-6. Taken together, it is possible that IL-6 promote SOX5 expression and thereby enhance the binding of SOX5 to RANKL, and the increase in SOX5 binding to the RANKL promoter at least in part account for the much larger increase in RANKL expression in RA SF.

IL-6 plays a key role in driving osteoclastogenesis and bone resorption in RA. Previous study indicate IL-6 does not induce osteoclast formation per se, but could mediated it by a direct effects in which IL-6 engages IL-6R directly on osteoclast precursors or by an indirect effect on prompting RANKL expression in mesenchymal cells^29^. Our data shed a new light on that IL-6 mediated regulation of inflammatory bone erosion in RA by an indirect effect on prompting transcriptional factor SOX5 binding to *RANKL*.

These results are further supported by our *in vivo* findings that local Sox5 gene silencing results in markedly bone erosion in CIA mice, which could be attributed in part to the concomitant *Rankl* down-regulation. RANKL plays a crucial role in osteoclast formation, function and survival. Our data indicate blocking endogenous Sox5 did not affect osteoclast formation in the presence with RANKL; however, Lv-siSox5 transfection is incapable of driving osteoclastogenesis in osteoclast precursor cell in the absence of RANKL, highlighting that SOX5 mediated bone erosion is depended of RANKL.

To date, transcriptional regulatory regions of mouse *Rankl* has been well studied, but the regulatory motifs in human *RANKL* remains largely unclear^30^,^31^. Our previous data showed that SOX5 could bind to the −1857 site of the *RANKL* promoter^7^. However, whether one or multiple SOX5 binding sites could regulate *RANKL* expression is beyond the scope of the current study. The transcriptional regulatory mechanism of SOX5 on *RANKL* should be further clarified in the future. Our data indicated that CIA incidence and arthritis score was decreased after Lv-siSox5 treatment. Although our data provide solid evidence that SOX5 is critically involved in RA bone erosion by modulating the RANKL pathway, we can’t exclude a potential anti-inflammatory role of SOX5 contribute to improved bone erosion in CIA in current study. Further study also need to clarify the effect of SOX5 on inflammation process in CIA mice.

In summary, our findings establish a key role for SOX5 in inflammation-mediated RANKL expression in arthritic joint. Given that extensive inhibit of RANKL expression could affect bone turnover, our study positions selective blockage of SOX5 as a potential novel therapeutic target for RA and possibly other bone resorptive diseases, such as osteoporosis.

## Additional Information

**How to cite this article**: Feng, X. *et al*. Modulation of IL-6 induced *RANKL* expression in arthritic synovium by a transcription factor SOX5. *Sci. Rep.* **6**, 32001; doi: 10.1038/srep32001 (2016).

## Supplementary Material

Supplementary Information

## References

[b1] Danks L. & Takayanagi H. Immunology and bone. J Biochem 154, 29–39 (2013). 23750028 10.1093/jb/mvt049

[b2] Choi Y., Arron J. R. & Townsend M. J. Promising bone-related therapeutic targets for rheumatoid arthritis. Nat Rev Rheumatol 5, 543–8 (2009). 19798028 10.1038/nrrheum.2009.175 PMC2797318

[b3] Cohen S. B. . Denosumab treatment effects on structural damage, bone mineral density, and bone turnover in rheumatoid arthritis: a twelve-month, multicenter, randomized, double-blind, placebo-controlled, phase II clinical trial. Arthritis Rheum 58, 1299–309 (2008). 18438830 10.1002/art.23417

[b4] Takayanagi H. New developments in osteoimmunology. Nat Rev Rheumatol 9, 684–9 (2012). 23070645 10.1038/nrrheum.2012.167

[b5] O’Brien C. A. Control of RANKL gene expression. Bone 46, 911–9 (2010). 19716455 10.1016/j.bone.2009.08.050 PMC2842447

[b6] Lotinun S. . Osteoclast-specific cathepsin K deletion stimulates S1P-dependent bone formation. J Clin Invest 123, 666–81 (2013). 23321671 10.1172/JCI64840 PMC3561821

[b7] Tan W. . A functional RANKL polymorphism associated with younger age at onset of rheumatoid arthritis. Arthritis Rheum 62, 2864–75 (2010). 20533289 10.1002/art.27589 PMC2944013

[b8] Wu H. . Interaction between RANKL and HLA-DRB1 genotypes may contribute to younger age at onset of seropositive rheumatoid arthritis in an inception cohort. Arthritis Rheum 50, 3093–103 (2004). 15476205 10.1002/art.20555

[b9] Rakhmanov M. . High levels of SOX5 decrease proliferative capacity of human B cells, but permit plasmablast differentiation. PLoS One 9, e100328 (2014). 24945754 10.1371/journal.pone.0100328 PMC4063782

[b10] Ikeda T. . Identification and characterization of the human long form of Sox5 (L-SOX5) gene. Gene 298, 59–68 (2002). 12406576 10.1016/s0378-1119(02)00927-7

[b11] Wunderle V. M., Critcher R., Ashworth A. & Goodfellow P. N. Cloning and characterization of SOX5, a new member of the human SOX gene family. Genomics 36, 354–8 (1996). 8812465 10.1006/geno.1996.0474

[b12] Lai T. . SOX5 controls the sequential generation of distinct corticofugal neuron subtypes. Neuron 57, 232–47 (2008). 18215621 10.1016/j.neuron.2007.12.023

[b13] Wegner M. All purpose Sox: The many roles of Sox proteins in gene expression. Int J Biochem Cell Biol 42, 381–90 (2010). 19631281 10.1016/j.biocel.2009.07.006

[b14] Huang D. Y. . Transcription factor SOX-5 enhances nasopharyngeal carcinoma progression by down-regulating SPARC gene expression. J Pathol 214, 445–55 (2008). 18085523 10.1002/path.2299

[b15] Ueda R., Yoshida K., Kawase T., Kawakami Y. & Toda M. Preferential expression and frequent IgG responses of a tumor antigen, SOX5, in glioma patients. Int J Cancer 120, 1704–11 (2007). 17230535 10.1002/ijc.22472

[b16] Wang D., Han S., Wang X., Peng R. & Li X. SOX5 promotes epithelial-mesenchymal transition and cell invasion via regulation of Twist1 in hepatocellular carcinoma. Med Oncol 32, 461 (2015). 25572815 10.1007/s12032-014-0461-2

[b17] Tanaka S. . Sox5 and c-Maf cooperatively induce Th17 cell differentiation via RORgammat induction as downstream targets of Stat3. J Exp Med 211, 1857–74 (2014). 25073789 10.1084/jem.20130791 PMC4144730

[b18] Arnett F. C. . The American Rheumatism Association 1987revised criteria for the classification of rheumatoid arthritis. Arthritis Rheum 31, 315–24 (1988). 3358796 10.1002/art.1780310302

[b19] Wu C. W. . Validation of American College of Rheumatology classification criteria for knee osteoarthritis using arthroscopically defined cartilage damage scores. Semin Arthritis Rheum 35, 197–201 (2005). 16325660 10.1016/j.semarthrit.2005.06.002

[b20] Bartok B., Hammaker D. & Firestein G. S. Phosphoinositide 3-kinase delta regulates migration and invasion of synoviocytes in rheumatoid arthritis. J Immunol 192, 2063–70 (2014). 24470496 10.4049/jimmunol.1300950

[b21] Rosengren S., Boyle D. L. & Firestein G. S. Acquisition, culture, and phenotyping of synovial fibroblasts. Methods Mol Med 135, 365–375 (2007). 17951672 10.1007/978-1-59745-401-8_24

[b22] Miyazawa K., Mori A. & Okudaira H. Establishment and characterization of a novel human rheumatoid fibroblast-like synoviocyte line, MH7A, immortalized with SV40 T antigen. J Biochem 124, 1153–62 (1998). 9832620 10.1093/oxfordjournals.jbchem.a022233

[b23] Wang F. . Interleukin-29 modulates proinflammatory cytokine production in synovial inflammation of rheumatoid arthritis. Arthritis Res Ther 14, R228 (2012). 23078630 10.1186/ar4067 PMC3580539

[b24] Lai Kwan L. Q., King Hung K. O., Zheng B. J. & Lu L. Local BAFF gene silencing suppresses Th17-cell generation and ameliorates autoimmune arthritis. Proc Natl Acad Sci USA 105, 14993–8 (2008). 18820032 10.1073/pnas.0806044105 PMC2567481

[b25] Hino S. . FAD-dependent lysine-specific demethylase-1 regulates cellular energy expenditure. Nat Commun 3, 758 (2012). 22453831 10.1038/ncomms1755 PMC3316891

[b26] Brand D. D. & Latham K. A. & Rosloniec E. F. Collagen-induced arthritis. Nat Protoc 2, 1269–75 (2007). 17546023 10.1038/nprot.2007.173

[b27] Schramm C. . Susceptibility to collagen-induced arthritis is modulated by TGFbeta responsiveness of T cells. Arthritis Res Ther 6, R114–R119 (2004). 15059274 10.1186/ar1039 PMC400430

[b28] de la Rocha A. M., Sampron N., Alonso M. M. & Matheu A. Role of SOX family of transcription factors in central nervous system tumors. Am J Cancer Res 4, 312–24 (2014). 25057435 PMC4106650

[b29] Axmann R. 1., Böhm C., Krönke G., Zwerina J., Smolen J. & Schett G. Inhibition of interleukin-6 receptor directly blocks osteoclast formation *in vitro* and *in vivo*. Arthritis Rheum 60, 2747–56 (2009). 19714627 10.1002/art.24781

[b30] Nerenz R. D., Martowicz M. L. & Pike J. W. An enhancer 20 kilobases upstream of the human receptor activator of nuclear factor-kappaB ligand gene mediates dominant activation by 1,25-dihydroxyvitamin D3. Mol Endocrinol 22, 1044–56 (2008). 18202151 10.1210/me.2007-0380 PMC2366191

[b31] Martin T. J. Historically significant events in the discovery of RANK/RANKL/OPG. World J Orthop 4, 186–97 (2013). 24147254 10.5312/wjo.v4.i4.186 PMC3801238

