# Supplementary material for: Modulation of IL-6 induced RANKL expression in arthritic synovium by a transcription factor SOX5
Source: Sci Rep. 2016 Aug 23;6:32001. doi: 10.1038/srep32001 (PMC4994074; doi:10.1038/srep32001)
Supplement: Supplementary Information [file srep32001-s1.doc]

**Modulation of IL-6 induced *RANKL* expression in arthritic synovium by a transcription factor SOX5**

Xiaoke Feng,^1,2^,^#^ Yumeng Shi,^1^,^#^ Lingxiao Xu,^1^ Qiuyue Peng,^2^ Fang Wang,^3^ Xiaoxi Wang,^1^ Wei Sun,^3^ Yan Lu,^3^ Betty P. Tsao,^4^ Miaojia Zhang,^1^ Wenfeng Tan^1^*

**Table S1**. Clinical characteristics of RA and OA patients

| Assessment | RA (*n* = 30) | OA (*n* = 27) |
| --- | --- | --- |
| Age, median (range) | 39 (23-58) | 49 (43-70) |
| Female, *n* (%) | 26 (86.7) | 24 (88.9) |
| Disease duration (yr), median (range) | 3 (0.5-6) | 9 (2-17) |
| Tender joint count, *n* (range) | 9 (3-22) | - |
| Swollen joint count, *n* (range) | 5 (4-15) | - |
| Patients global assessment (mm), median (range) | 60 (45-100) | - |
| ESR (mm/h), median (range) | 58 (35-110) | - |
| CRP (mg/dl), median (range) | 12.10 (1.02-30.24) | - |
| DAS 28-joint assessment, median (range) | 5.61 (4.63-7.11) | - |
| RF positivity, *n* (%) | 27 (90) | - |
| Anti-CCP antibody positivity, *n* (%) | 26 (86.7) | - |

*Patients who fulfilled the 2010 ACR/EULAR criteria for rheumatoid arthritis were included. Anti-CCP, anti-cyclic citrullinated peptide; CRP, C-reactive protein; DAS, disease activity score; ESR, erythrocyte sedimentation rate; RF, rheumatoid factor

**Table S2. siRNA sequences**

| Species | Strand | Sequences |
| --- | --- | --- |
| Mouse *SOX5* | Sense  Antisense | 5’-GATCCCGCTCCATACAACTCATCTATTGATATCCGTAGATGAGTTGTATGGAGCTTTTTTCCAAA-3’  5’- AGCTTTTGGAAAAAAGCTCCATACAACTCATCTACGGATATCAATAGATGAGTTGTATGGAGCGG-3’ |
| Human *SOX5* | Sense  Antisense | 5’- GATCCCGAGCACTTACGGTGTGAAATTGATATCCGTTTCACACCGTAAGTGCTCTTTTTTCCAAA-3’  5’- AGCTTTTGGAAAAAAGAGCACTTACGGTGTGAAACGGATATCAATTTCACACCGTAAGTGCTCGG-3’ |
| Control siRNA | Sense  Antisense | 5’-CCUACGCCACCAAUUUCGU-3’  5’-ACGAAAUUGGUGGCGUAGG-3’ |

**Table S3.** PCR primer

| Genes | Forward | Reverse |
| --- | --- | --- |
| *SOX5* | CAGCCAGAGTTAGCACAATAGG | CTGTTGTTCCCGTCGGAGTT |
| RANKL  (for chip PCR) | TGAGGTTGAAGTAATCAAGCCA | GATGTGAGACAAATCCCAGGCT |

**Figure S1**


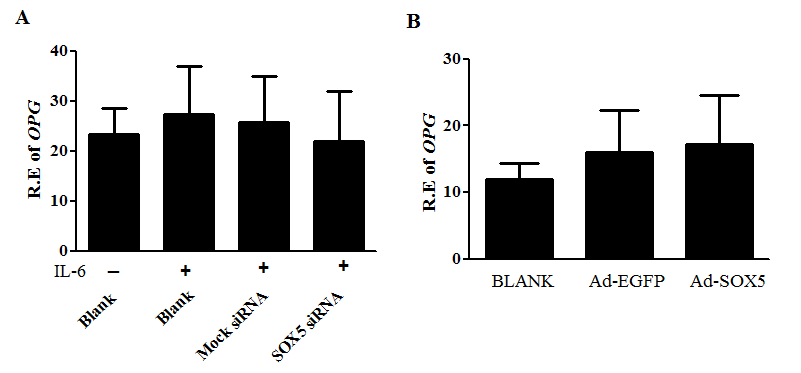


Figure S1. OPG mRNA expression in SOX5 knockdown or overexpression MH7A. A, IL-6 induced OPG expression in SOX5-shRNA treated or mock-transfected MH7A. B, Expression of SOX5 in MH7A after Ad-SOX5 transfection.

**Figure S2**


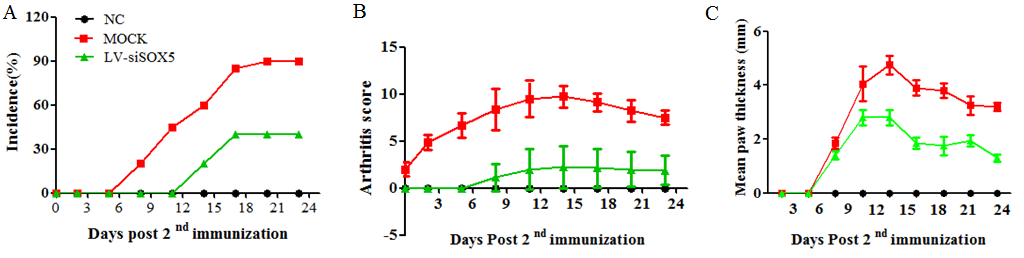


Figure S2. Cumulative incidence of arthritis development (A), arthritis score (B) and articular swelling in normal mice and CIA mice with intra-articular injection of LV-siSox5 or Mock siRNA.
